# Predictors of Prolonged Intensive Care Unit Stay and In-Hospital Mortality Following Cardiac Surgery: An Integrated Analysis from the PROCARD-ATI Study

**DOI:** 10.3390/jcm14082747

**Published:** 2025-04-16

**Authors:** Călin-Dinu Hădăreanu, Diana-Ruxandra Hădăreanu, Flavia-Mihaela Stoiculescu, Mihaela-Corina Berceanu, Ionuț Donoiu, Octavian Istrătoaie, Cristina Florescu, Marius-Bogdan Novac, Victor-Cornel Raicea

**Affiliations:** 1 Doctoral School, University of Medicine and Pharmacy of Craiova, 2 Petru Rares St., 200349 Craiova, Romania; 2 Department of Cardiovascular Surgery, Clinical Emergency County Hospital of Craiova, 1 Tabaci St., 200642 Craiova, Romania; 3 Department of Cardiology, University of Medicine and Pharmacy of Craiova, 2 Petru Rares St., 200349 Craiova, Romania; ionut.donoiu@umfcv.ro (I.D.);; 4 Department of Cardiology, Clinical Emergency County Hospital of Craiova, 1 Tabaci St., 200642 Craiova, Romania; 5 Department of Cardiology, Filantropia Clinical Hospital of Craiova, 28 Sararilor St., 200516 Craiova, Romania; 6 Department of Anesthesiology and Intensive Care, University of Medicine and Pharmacy of Craiova, 2 Petru Rares St., 200349 Craiova, Romania

**Keywords:** cardiac surgery, prognosis, intensive care unit stay, in-hospital mortality, aortic cross-clamp time, cardiopulmonary bypass time

## Abstract

**Background:** Despite advances in surgical techniques and perioperative management, reliable intraoperative predictors of adverse postoperative outcomes in cardiac surgery remain elusive. This study aimed to identify perioperative factors associated with prolonged intensive care unit (ICU) stay and in-hospital mortality while defining actionable thresholds. **Methods**: A retrospective analysis was conducted on 130 adult cardiac surgery patients (with a median age of 61 years, 66.2% men) from October 2022 to November 2024. Data on preoperative risk factors, intraoperative variables (cardiopulmonary bypass time-CPBT, aortic cross-clamp time-AXCT), and postoperative outcomes (ICU length of stay, in-hospital mortality) were extracted from electronic medical records. **Results**: Prolonged ICU stay (≥7 days) occurred in 38.5% of patients, and in-hospital mortality was 10%. AXCT was the sole independent predictor of prolonged ICU stay (OR = 1.046, 95% CI = 1.014–1.080, *p* = 0.005), with a 110-min cut-off (sensitivity 71%, specificity 61%, AUC = 0.729). A Kaplan–Meier analysis showed significantly longer ICU stays above this threshold (*p* = 0.006). For in-hospital mortality, prolonged CPBT (OR = 1.030, 95% CI = 1.003–1.057, *p* = 0.030), emergency surgery (OR = 0.043, 95% CI = 0.002–0.863, *p* = 0.040), and higher AXCT (OR = 0.965, 95% CI = 0.934–0.997, *p* = 0.034) were the independent predictors. A receiver operating characteristic analysis identified 140 min for AXCT (sensitivity 67%, specificity 70%, AUC = 0.707) and 227 min for CPBT (sensitivity 83%, specificity 69%, AUC = 0.824) as the optimal cut-offs. A combined model (emergency surgery yes/no, AXCT > 140 min, CPBT > 227 min) yielded excellent discrimination (AUC = 0.846). **Conclusions**: These findings suggest perioperative benchmarks that may guide surgical teams in refining operative strategies, reducing ICU resource utilization, and improving survival following cardiac surgery.

## 1. Introduction

Cardiac surgery remains a cornerstone treatment for advanced cardiovascular disease; however, postoperative outcomes—particularly intensive care unit (ICU) stay and in-hospital mortality—are critical indicators of patient recovery and healthcare resource utilization. Prolonged ICU stays have been documented in 4% to 11% of cardiac surgery patients, with some studies reporting rates as high as 36% [1]. Notably, a large study of 252,948 cardiac surgery patients from 101 hospitals demonstrated a U-shaped relationship between ICU stay and hospital mortality [2]. Such extended ICU stays not only strain critical care resources—leading to higher costs, canceled procedures, and longer waiting lists—but also correlate with increased morbidity and cause significant distress for patients and families [3], reducing their overall quality of life [1]. Meanwhile, in-hospital mortality serves as a vital marker of surgical quality and patient safety [4].

Despite continuous advancements in surgical techniques and perioperative management, the identification of reliable intraoperative predictors that can forecast adverse postoperative outcomes remains challenging. Several factors—ranging from preoperative comorbidities to intraoperative variables—have been implicated in influencing outcomes due to their roles in inducing myocardial ischemia and systemic inflammatory responses [5]. However, explicit intraoperative thresholds that can be directly applied in clinical practice remain elusive. Moreover, recent advances in predictive modeling have provided important insights into risk stratification before cardiac surgery. The European Multicenter Study on Coronary Artery Bypass Grafting (E-CABG) registry [6] analyzed over 7000 patients undergoing isolated coronary artery bypass grafting to develop a nomogram incorporating ten preoperative factors to predict prolonged ICU stay. Although such preoperative models are valuable for early risk assessments, their exclusive reliance on preoperative data limits the potential for real-time intraoperative decision-making. Existing models largely focus on estimating short-term mortality risk, with the European System for Cardiac Operative Risk Evaluation (EuroSCORE) [7] and the Society of Thoracic Surgeons (STS) score [8,9] being among the most widely used.

Different models for prolonged ICU stay were validated, demonstrating that models originally designed to predict mortality (e.g., the Parsonnet and EuroSCORE models) can effectively identify patients at risk [10]. Moreover, some studies achieved high accuracy in predicting operative mortality for procedures without established risk scores [11], while others have applied machine learning techniques that outperformed conventional risk scores in predicting prolonged ICU stay [12]. Furthermore, research using both preoperative and intraoperative variables from large databases has demonstrated that accurate prediction is achievable, with only minimal loss of accuracy when restricted to preoperative factors [13]. Finally, in another population-based cohort, pragmatic clinical models showed excellent discrimination for hospital length of stay [14]. Collectively, these findings underscore the potential of integrating both preoperative and intraoperative factors for improved risk stratification in cardiac surgery—a gap that the present study aims to address by focusing on real-time intraoperative metrics.

Accordingly, the aim of this study was to evaluate intraoperative and perioperative factors as predictors of prolonged ICU stay and in-hospital mortality in patients undergoing cardiac surgery while also defining perioperative thresholds that could improve postoperative outcomes.

## 2. Materials and Methods

### 2.1. Study Population and Data Collection

**Figure 1:**
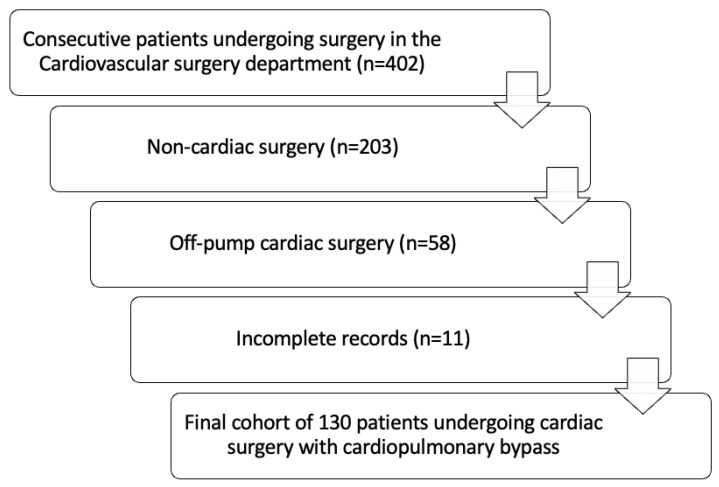
Flowchart describing the patient selection process.

We retrospectively reviewed the records of all consecutive patients aged 18 years and older who underwent surgery at the Cardiovascular Surgery Department of the Emergency Clinical County Hospital of Craiova, Romania, between October 2022 and November 2024. The inclusion criteria included patients who underwent cardiac surgery with cardiopulmonary bypass. The exclusion criteria included patients with incomplete records or those undergoing either off-pump surgery or procedures for non-cardiac indications (Figure 1). Data were extracted from the hospital’s electronic medical records using a standardized data collection form. The collected variables included demographic data, preoperative risk factors, pre- and postoperative biochemical and echocardiographic data, as well as intraoperative variables—cardiopulmonary bypass time (CPBT) and aortic cross-clamp time (AXCT). The primary outcome was defined as prolonged ICU stay (more than the median value of the study cohort), and the secondary outcome was in-hospital mortality (all-cause death during the same hospital admission).

### 2.2. Statistical Analysis

Descriptive statistics were computed for all variables. The distribution of the continuous variables was assessed using the Shapiro–Wilk test. The continuous variables were presented as means ± standard deviations (if normally distributed) or medians with interquartile ranges (IQRs) (if non-normally distributed), while the categorical variables were expressed as frequencies and percentages. To compare the continuous variables between groups, we used the Student’s *t*-test for normally distributed data or the Mann–Whitney U test for non-normally distributed data. The chi-square test was employed for the categorical comparisons. A univariate logistic regression analysis was conducted to identify factors associated with prolonged ICU stay and in-hospital mortality. Variables with a *p*-value < 0.05 were then entered into multivariable logistic regression models to determine the independent predictors. To evaluate the discriminative ability of significant predictors (area under the curve, or AUC) and derive optimal cut-off values, we performed a receiver operating characteristic (ROC) analysis and Youden’s J index. The Kaplan–Meier survival analysis was used to compare outcomes based on these cut-offs, and the differences between the groups were analyzed using the log-rank test. Additionally, a multivariable model for the secondary outcome incorporating binary predictors based on the independent predictors and cut-off values (yes/no or </≥ than the cut-off) was constructed. The predicted probabilities from this model were used to generate an ROC curve for the overall discrimination. The statistical analysis was performed using SPSS version 23 for Mac (SPSS Inc., IBM Co., Chicago, IL, USA), and a two-sided *p*-value < 0.05 was considered significant.

## 3. Results

Of the 402 patients screened, a total of 130 patients (median age = 61, IQR of 56–69, 66.2% men) met the inclusion criteria. The baseline demographic and clinical characteristics, as well as key perioperative data, are detailed in Table 1. Most patients underwent surgery for valvular heart disease (53, 40.8%), followed by coronary artery bypass grafting (33, 25.4%). The median ICU stay was 6 days (IQR = 4–8 days), while in-hospital mortality occurred in 10% of cases. Patients with a prolonged ICU stay (≥7 days) had significantly longer overall hospital stays (*p* < 0.001), higher in-hospital mortality rates (*p* = 0.032), a greater prevalence of chronic obstructive pulmonary disease (*p* = 0.008), elevated preoperative creatine kinase levels (*p* = 0.022), longer CPBT and AXCT (both *p* < 0.001), higher postoperative neutrophil counts (*p* = 0.032), and lower postoperative thrombocyte counts (*p* = 0.010).

In the univariate analysis, prolonged ICU stay was significantly associated with longer CPBT (OR = 1.011, IQR = 1.005–1.016, *p* < 0.001), extended AXCT (OR = 1.014, 95% CI = 1.007–1.022, *p* < 0.001), the need for temporary pacing, smoking (OR = 8.942, 95% CI = 1.126–71.045, *p* = 0.038), chronic obstructive pulmonary disease (OR = 0.137, 95% CI = 0.026–0.722, *p* = 0.019), postoperative thrombocyte (OR = 0.990, 95% CI = 0.981–0.998, *p* = 0.019) and neutrophil (OR = 1.157, 95% CI = 1.010–1.324, *p* = 0.035) counts, and an increase in creatinine relative to baseline (OR = 5.978, 95% CI = 1.336–26.752, *p* = 0.019, Table 2). However, the multivariable logistic regression analysis revealed that only AXCT remained an independent predictor of prolonged ICU stay (OR = 1.046, 95% CI = 1.014–1.080, *p* = 0.005, Table 3).

**Figure 2:**
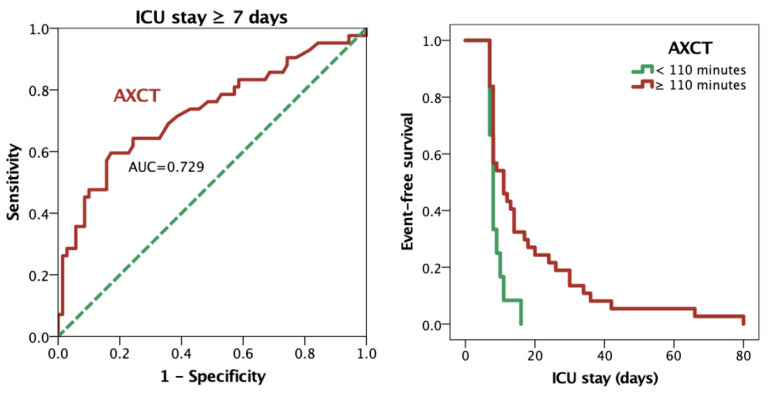
Receiver operating characteristic curve for aortic cross-clamp time in predicting an ICU stay of ≥7 days (**left**). Kaplan–Meier curve comparing ICU stay for patients with aortic cross-clamp time < 110 min versus ≥ 110 min (**right**). Abbreviations: AUC, area under the curve; AXCT, aortic cross-clamp time; ICU, intensive care unit.

The ROC analysis demonstrated that an AXCT cut-off of 110 min best predicted prolonged ICU stay, with a sensitivity of 71%, a specificity of 61%, and an AUC of 0.729 (Figure 2). The Kaplan–Meier survival analysis further confirmed that patients with AXCTs exceeding 110 min had a significantly higher probability of remaining in the ICU for ≥7 days (log-rank *p* = 0.006, Figure 2).

For in-hospital mortality, the univariate analysis identified several significant predictors, including emergency surgery (OR = 0.082, 95% CI = 0.022–0.298, *p* < 0.001), CPBT (OR = 1.012, 95% CI = 1.005–1.019, *p* = 0.001), AXCT (OR = 1.010, 95% CI = 1.001–1.018, *p* = 0.021), vasopressor use at ICU admission (IQR = 0.154, 95% CI = 0.039–0.603, *p* = 0.007), and ICU length of stay (OR = 1.095, 95% CI = 1.035–1.158, *p* = 0.001, Table 4). In the multivariable analysis, prolonged CPBT (OR = 1.030, 95% CI = 1.003–1.057, *p* = 0.030), emergency surgery (OR = 0.043, 95% CI = 0.002–0.863, *p* = 0.040), and increased AXCT (OR = 0.965, 95% CI = 0.934–0.997, *p* = 0.034) emerged as independent predictors of in-hospital mortality (Table 5).

**Figure 3:**
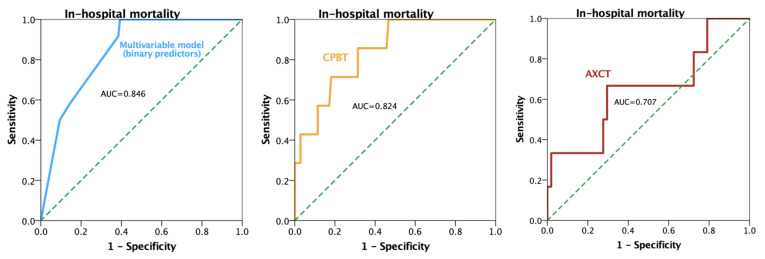
Receiver operating characteristic curve for predictors of in-hospital mortality. Abbreviations: AUC, area under the curve; AXCT, aortic cross-clamp time; CPBT, cardiopulmonary bypass time.

The ROC analysis for in-hospital mortality indicated an AXCT cut-off of 140 min (sensitivity of 67%, specificity of 70%, AUC = 0.707) and a CPBT cut-off of 227 min (sensitivity of 83%, specificity of 69%, AUC = 0.824) for the outcome prediction (Figure 3). Moreover, a multivariable model incorporating the binary predictors of emergency surgery (yes/no), CPBT > 227 min, and AXCT > 140 min yielded an AUC of 0.846, demonstrating excellent discriminative ability (Figure 3).

## 4. Discussion

### 4.1. Key Findings

Our study evaluated intraoperative and perioperative factors as predictors of prolonged ICU stay and in-hospital mortality following cardiac surgery. Significant differences in baseline, intraoperative, and postoperative variables were found between patients with prolonged ICU stays and those without. In the univariate analysis, several factors—including longer CPBT, extended AXCT, temporary pacing, smoking, chronic obstructive pulmonary disease, and postoperative laboratory abnormalities—were associated with prolonged ICU stay; however, after adjustment for confounders, only AXCT remained an independent predictor. Furthermore, the optimal threshold for AXCT to predict prolonged ICU stay (≥7 days) was 110 min, with a sensitivity of 71%, a specificity of 61%, and an AUC of 0.729. Patients with AXCTs exceeding 110 min experienced significantly longer ICU stays (*p* = 0.006).

### 4.2. Predictors of Prolonged ICU Stay

However, in previous research, several surgical characteristics have been consistently linked to ICU stay, including CPBT and blood transfusion [15]. Several other parameters were found to be independent predictors of the length of ICU stay in a systematic review including 29 papers—older age, chronic obstructive pulmonary disease, renal failure or dysfunction, atrial fibrillation, low ejection fraction, NYHA class III–IV, non-elective surgery, prior cardiac surgery, and inotropic support [5]. In contrast, our analysis found that only AXCT remained an independent predictor of prolonged ICU stay, rather than CBPT. One potential explanation is that AXCT may more directly reflect the duration of myocardial ischemia and subsequent inflammatory responses, having a critical role in postoperative recovery and, thus, overshadowing the influence of total CPBT in the final multivariable model. Nonetheless, our results closely align with those reporting that longer AXCT is significantly associated with increased ICU stay [16].

Interestingly, older age did not emerge as an independent predictor of prolonged ICU stay or in-hospital mortality in our cohort, despite previous findings identifying it as a significant risk factor [5]. Although age is traditionally considered influential in cardiac surgery outcomes, it may have been overshadowed by stronger intraoperative factors—particularly AXCT—that more directly indicate myocardial ischemia and surgical stress. Additionally, older patients with significant comorbidities might have received heightened perioperative care, mitigating age-related risk.

The relationship between AXCT and ICU length of stay has also been investigated in other studies. For instance, a study on a large cohort of patients concluded that longer AXCT was associated with a higher likelihood of prolonged postoperative mechanical ventilation and hospital stay [17]. Similarly, AXCT, along with other factors, was found to be strongly related to extended ICU stays [18], and prolonged ICU stays were linked to worse overall outcomes and higher in-hospital mortality [19]. In our study, we observed that prolonged AXCT was significantly associated with increased ICU stays, which aligns with these findings. However, our multivariable analysis ultimately identified AXCT as the only independent predictor of prolonged ICU stay. One possible explanation is that AXCT more directly reflects myocardial ischemic duration, since a direct linear correlation with postoperative troponin I levels has been demonstrated—indicating that even modest increases in AXCT could result in greater myocardial injury [20], overshadowing the impact of total CPBT when controlling for other variables.

In our analysis, a longer AXCT was strongly associated with prolonged ICU stay, likely reflecting extended myocardial ischemia that delays recovery. Conversely, when controlling for CPBT, AXCT showed an inverse relationship with in-hospital mortality. This paradox may arise from surgical strategies in complex cases, where surgeons deliberately minimize AXCT to reduce ischemic injury while accepting a longer CPBT. These factors may mask AXCT’s independent contribution once CPBT—a strong mortality predictor—is taken into account, thereby explaining the differing role of AXCT in predicting ICU stay versus in-hospital mortality.

### 4.3. Predictors of Mortality

Several studies have examined the relationship between AXCT and postoperative outcomes. Prolonged AXCT was reported as an independent predictor of postoperative morbidity and mortality in a large cohort of patients undergoing aortic valve replacement [21]. Similarly, another analysis demonstrated that extended AXCT was significantly associated with major complications and mortality—especially when it exceeded 90 min [22]—or found that, in complex cardiac surgeries, very long AXCT durations (over 300 min) were linked to significant early mortality and morbidity [23].

A large-scale study on adult cardiac surgery patients demonstrated that both AXCT and CPBT were strong predictors of 30-day postoperative mortality, identifying 150 min for AXCT and 240 min for CPBT as the optimal cut-offs [24]. In comparison, our findings similarly highlighted prolonged CPBT, increased AXCT, and emergency surgery as independent predictors of in-hospital mortality, but with slightly different thresholds as follows: 140 min for ACCT (sensitivity of 67%, specificity of 70%, AUC = 0.707) and 227 min for CPBT (sensitivity of 83%, specificity of 69%, AUC = 0.824). Moreover, when these binary predictors (emergency surgery yes/no, CPBT > 227 min, and aortic cross-clamp time > 140 min) were combined in a single model, the discriminative ability was excellent (AUC = 0.846). These convergent results emphasize that once the operative times exceed certain time-based benchmarks, the risk of adverse outcomes rises markedly, although the precise cut-off can vary with differences in patient populations, procedural complexity, and myocardial protection strategies.

The discrepancies in thresholds for predicting either prolonged ICU stay or in-hospital mortality for AXCT and CBPT may be explained by our inclusion of a heterogeneous cardiac surgical population, which incorporated multiple types of procedures rather than focusing solely on isolated coronary artery bypass grafting or valve surgeries. Such diversity likely necessitates longer operative times due to the increased complexity of concomitant interventions and varying myocardial protection strategies across institutions. Additionally, differences in patient demographics and postoperative management practices may also contribute to the higher threshold observed in our analysis.

In addition, while larger multicenter studies have developed predictive models for prolonged ICU stay, such as the nomogram derived from the E-CABG registry [6], which analyzed 7352 isolated coronary artery bypass grafting patients and incorporated ten preoperative factors, the translation of these models into practical clinical tools remains challenging. In contrast, our single-center analysis identifies actionable intraoperative thresholds that can be monitored in real time to guide surgical strategy and ICU resource planning. Moreover, emergency procedures, which are associated with higher early mortality and could require separate stratification, were included to reflect real-world practice. Integrating these intraoperative parameters with existing preoperative models may, thus, further refine risk stratification and enhance clinical decision-making, especially in a heterogeneous surgical population that includes both elective and emergency cases.

### 4.4. Clinical Perspective

Overall, our study not only confirms that extended intraoperative durations are linked to adverse postoperative outcomes but also extends the current literature by establishing specific perioperative thresholds that can be applied across a heterogeneous patient population. By providing actionable intraoperative benchmarks, our findings offer valuable insights for optimizing surgical strategies, improving resource allocation in the cardiac ICU, and, ultimately, enhancing patient survival.

A major novel aspect of our study is the integrated approach used to derive precise intraoperative thresholds, which can serve as direct targets for operative management. Specifically, maintaining AXCT below 110 min may reduce the ICU stay duration, while keeping it below 140 min—along with limiting CPBT to under 227 min—could lower in-hospital mortality. These actionable benchmarks not only confirm that extended intraoperative durations are linked to adverse postoperative outcomes but also extend the current literature by establishing perioperative thresholds that can be applied across a heterogeneous patient population. Moreover, our study’s value is enhanced by its inclusive design; rather than focusing on isolated procedures, we encompassed various cardiac surgeries, thereby improving the external validity and offering broader insights for optimizing care delivery, resource allocation, and, ultimately, improving patient survival.

### 4.5. Limitations

We acknowledge the several limitations of our study. Firstly, its single-center, retrospective design and relatively small sample size may limit the generalizability of our findings compared to larger, multicenter registries. Secondly, although our analysis provides actionable intraoperative thresholds, these variables are inherently subject to interoperator variability and may be influenced by institutional practices. Thirdly, our cohort included both elective and emergency procedures without separate stratification, which may mask the impact of emergency surgery on the outcomes. Additionally, we did not perform a stratified analysis for emergency procedures or a competing-risk analysis, excluding early deaths, when evaluating ICU length of stay. Although such analyses could offer additional insights into the distinct effects of intraoperative variables in emergency versus elective cases and help mitigate bias from early mortality, the limited sample size in the emergency subgroup posed a significant risk of overfitting. Lastly, while our study highlights the value of intraoperative variables, it does not incorporate a comprehensive preoperative risk model, and, as such, our findings should be interpreted in conjunction with established preoperative predictors.

## 5. Conclusions

In conclusion, our study demonstrates that among various intraoperative and perioperative factors, aortic cross-clamp time is a key predictor of prolonged ICU stay, with a cut-off of 110 min being the most predictive of an ICU stay ≥7 days. Additionally, prolonged CPBT, emergency surgery, and increased AXCT are independently associated with in-hospital mortality, with optimal thresholds of 140 min for AXCT and 227 min for CPBT. A combined multivariable model using these binary predictors yielded excellent discriminative ability (AUC = 0.846) for predicting in-hospital mortality. These actionable benchmarks can potentially guide surgical teams in refining their techniques to reduce ICU resource utilization and improve patient survival following cardiac surgery.

## Figures and Tables

**Table 1:** Characteristics of the study population and comparison between patients with ICU stay </≥ 7 days.

Parameter	Entire Cohort (*n* = 130)	ICU Stay < 7 Days (*n* = 80)	ICU Stay ≥ 7 Day (*n* = 50)	*p* Value
ICU length of stay (days)	6 [4–8]	4 [3–6]	9 [8–17]	<0.001
Hospitalization duration (days)	19 [14–27]	15 [12–21]	23 [20–35]	<0.001
In-hospital death	13 (10%)	4 (5%)	9 (18%)	0.032
Emergency surgery	20 (15.4%)	8 (10%)	12 (24%)	0.053
Type of Surgery				0.408
Coronary artery bypass grafting	33 (25.4%)	20 (25%)	13 (26%)	
Valve disease	53 (40.8%)	32 (40%)	21 (42%)	
Other	31 (23.8%)	19 (24%)	12 (24%)	
Combined interventions	13 (10%)	9 (11%)	4 (8%)	
Demographic				
Age (years)	61 [56–69]	60 [56–69]	65 [56–70]	0.120
Body mass index (kg/m^2^)	27.5 ± 4.6	27.8 ± 4.5	27.1 ± 4.8	0.445
Sex (men)	86 (66.2%)	57 (71%)	29 (58%)	0.132
Risk factors				
Arterial hypertension	87 (66.9%)	58 (72.5%)	29 (58%)	0.887
NYHA class III/IV	26 (20%)	12 (13.5%)	14 (28%)	0.088
Type 2 Diabetes Mellitus	33 (25.4%)	25 (31%)	8 (16%)	0.181
Smoking	16 (12.3%)	15 (17%)	1 (2%)	0.015
Chronic obstructive pulmonary disease	8 (6.2%)	2 (2.5%)	6 (12%)	0.008
Stage 3–5 chronic kidney disease	9 (6.9%)	5 (6%)	4 (8%)	0.432
Preoperative				
Atrial fibrillation	19 (14.6%)	14 (16%)	5 (10%)	0.492
Left ventricular ejection fraction (%)	50 [45–55]	50 [50–55]	55 [50–55]	0.381
Tricuspid annulus plane systolic excursion (mm)	20 [19–22]	20 [20–21]	20 [19–22]	0.504
Systolic pulmonary artery pressure (mmHg)	20 [17–24]	29 [25–42]	26 [24–28]	0.053
Albumin (g/dL)	3.3 ± 0.6	3.3 ± 0.5	3.1 ± 0.8	0.230
Direct bilirubin (mg/dL)	0.3 [0.2–0.46]	0.30 [0.20–0.49]	0.31 [0.17–0.40]	0.480
Total bilirubin (mg/dL)	0.81 [0.57–1.33]	0.80 [0.55–1.30]	0.88 [0.60–1.42]	0.665
Alanine aminotransferase (U/L)	24 [18–42]	23 [18–43]	28 [19–39]	0.491
Aspartate aminotransferase (U/L)	28 [20–44]	30 [21–44]	27 [20–40]	0.602
Hemoglobin (g/dL)	13.8 [12.6–14.7]	13.9 [13.2–14.9]	13.7 [12.3–14.3]	0.212
Thrombocytes (n/uL)	215 ± 72 × 103	220 ± 73 × 103	208 ± 70 × 103	0.455
Leucocytes (n/uL)	7800 [6180–9826] × 103	7700 [6090–9400] × 103	8125 [6252–10,695] × 103	0.452
Lymphocytes (n/uL)	2000 [1590–2500] × 103	2000 [1680–2600] × 103	1880 [1325–2467] × 103	0.185
Neutrophils (n/uL)	5060 [3570–6480] × 103	5070 [3520–6540] × 103	5050 [3640–6300] × 103	0.957
Serum potassium (mmol/L)	4.4 [4–4.7]	4.4 [4–4.7]	4.5 [4.2–4.6]	0.519
Serum sodium (mmol/L)	140 [138–141]	140 [137–141]	140 [138–141]	0.778
Errythrocyte sedimentation rate (mm/h)	8 [5–23]	8 [3–19]	10 [5–39]	0.310
Fibrionogen (mg/dL)	338 [304–390]	342 [302–391]	338 [297–380]	0.630
C-reactive protein (mg/L)	3.37 [1.8–6]	3.3 [1.7–6]	3.9 [2–6]	0.839
Creatinin (mg/dL)	0.93 [0.81–1.08]	0.92 [0.8–1.07]	0.95 [0.81–1.16]	0.746
Urea (mg/dL)	39 [29–49]	38 [27–49]	40 [31–51]	0.467
NT-proBNP (pg/mL)	873 [198–1775]	765 [142–2829]	873 [475–1505]	0.450
Creatine kinase (U/L)	583 [342–810]	633 [461–820]	385 [66–648]	0.022
Intraoperative				
Cardiopulmonary bypass time (minutes)	185 [150–249]	170 [144–219]	245 [177–354]	<0.001
Aortic cross-clamp time (minutes)	111 [86–164]	104 [81–124]	149 [106–223]	<0.001
Postoperative				
Vasopressors at ICU admission	47 (36.2%)	24 (30%)	23 (46%)	0.065
Inotropes at ICU admission	51 (39.3%)	28 (35%)	23 (46%)	0.211
Left ventricular ejection fraction (%)	50 [45–55]	50 [45–55]	50 [45–50]	0.379
Systolic pulmonary artery pressure (mmHg)	24 [20–31]	20 [20–25]	20 [19–24]	0.649
Albumin (g/dL)	3.2 ± 0.4	3.3 ± 0.4	3.1 ± 0.4	0.065
Direct bilirubin (mg/dL)	0.5 [0.3–1.16]	0.43 [0.29–1.13]	0.6 [0.39–1.2]	0.227
Total bilirubin (mg/dL)	1 [0.7–2.1]	0.9 [0.65–2]	14 [0.83–2.59]	0.092
Alanine aminotransferase (U/L)	31 [20–52]	29 [20–47]	36 [22–77]	0.348
Aspartate aminotransferase (U/L)	61 [38–95]	59 [35–80]	67 [49–129]	0.089
Hemoglobin (g/dL)	9.4 [8.4–10.5]	9.4 [8.4–10.5]	9.1 [8.6–10.4]	0.708
Thrombocytes (n/uL)	152 [91–179] × 103	160 [110–188] × 103	108 [77–159] × 103	0.010
Leucocytes (n/uL)	11,000 [8475–13,450] × 103	10,080 [8400–12,750] × 103	11,600 [8450–15,100] × 103	0.176
Lymphocytes (n/uL)	900 [600–1600] × 103	900 [600–1600] × 103	800 [600–1600] × 103	0.431
Neutrophils (n/uL)	8750 [6900–10,800] × 103	8350 [6467–10,600] × 103	9650 [7922–13,225] × 103	0.032
Serum potassium (mmol/L)	4 [3.7–4.4]	4 [3.7–4.3]	4.2 [3.7–4.5]	0.288
Serum sodium (mmol/L)	141 [138–143]	141 [138–143]	142 [140–144]	0.293
Errythrocyte sedimentation rate (mm/h)	19.5 [7.3–33.3]	24 [9–40]	16.5 [7–25.5]	0.096
Fibrionogen (mg/dL)	507 [412–562]	524 [433–589]	452 [366–530]	0.053
C-reactive protein (mg/L)	121 [56–196]	123 [56–199]	119 [55–198]	0.918
Creatinin (mg/dL)	0.95 [0.73–1.23]	0.9 [0.71–1.19]	1.04 [0.79–1.58]	0.089
Urea (mg/dL)	40 [31–51]	39 [29–49]	42 [32–59]	0.152
NT-proBNP (pg/mL)	2915 [1948–5110]	2628 [2146–3379]	4466 [1540–11,152]	0.174
Creatine kinase (U/L)	620 [398–1262]	523 [318–944]	719 [504–1621]	0.067

Abbreviations: ICU, intensive care unit; NT-proBNP, *N*-terminal prohormone of brain natriuretic peptide; NYHA, New York Heart Association.

**Table 2:** Univariate logistic regression analysis for ICU stay ≥ 7 days.

Parameter	OR [95% CI]	*p* Value
Demographic and Clinical		
Age	1.019 [0.983–1.058]	0.306
Body mass index	0.970 [0.896–1.049]	0.442
Sex	1.795 [0.855–3.767]	0.122
Arterial hypertension	0.929 [0.334–2.578]	0.887
Type 2 Diabetes Mellitus	0.533 [0.211–1.349]	0.533
Smoking	8.942 [1.126–71.045]	0.038
Chronic obstructive pulmonary disease	0.137 [0.026–0.722]	0.019
Atrial fibrillation	1.476 [0.484–4.506]	0.494
Emergency surgery	0.384 [0.142–1.035]	0.058
Preoperative		
Left ventricular ejection fraction	1.016 [0.952–1.086]	0.627
Tricuspid annulus plane systolic excursion	1.052 [0.970–1.142]	0.221
Systolic pulmonary artery pressure	0.942 [0.879–1.010]	0.092
Albumin	0.582 [0.241–1.406]	0.229
Direct bilirubin	0.342 [0.045–2.599]	0.300
Total bilirubin	1.365 [0.747–2.496]	0.312
Alanine transaminase	1.006 [0.995–1.016]	0.292
Aspartate transaminase	1.002 [0.997–1.006]	0.447
Hemoglobin	1.000 [1.000–1.000]	0.451
Thrombocytes	0.998 [0.992–1.004]	0.451
Leucocytes	1.111 [0.969–1.275	0.131
Lymphocytes	0.751 [0.392–1.439]	0.388
Neutrophils	0.984 [0.915–1.058]	0.661
Serum potassium	0.992 [0.562–1.751]	0.978
Serum sodium	0.605 [0.935–1.040]	0.605
Errythrocyte sedimentation rate	1.048 [0.987–1.112]	0.126
Fibrionogen	0.999 [0.994–1.004]	0.634
C-reactive protein	0.974 [0.923–1.029]	0.349
Creatinin	0.832 [0.488–1.416]	0.497
Urea	1.005 [0.981–1.031]	0.675
NT-proBNP	1.000 [0.999–1.000]	0.951
Creatine kinase	1.000 [0.998–1.001]	0.415
Intraoperative		
Cardiopulmonary bypass time	1.011 [1.005–1.016]	<0.001
Aortic cross-clamp time	1.014 [1.007–1.022]	<0.001
Postoperative		
Vasopressors at ICU admission	0.503 [0.242–1.048]	0.066
Inotropes at ICU admission	1.582 [0.769–3.255]	0.213
Left ventricular ejection fraction	0.966 [0.867–1.076]	0.527
Systolic pulmonary artery pressure	0.892 [0.670–1.186]	0.431
Albumin	0.339 [0.106–1.091]	0.070
Direct bilirubin	0.967 [0.836–1.118]	0.648
Total bilirubin	1.273 [0.887–1.827]	0.190
Alanine transaminase	1.001 [0.999–1.004]	0.320
Aspartate transaminase	1.000 [0.999–1.000]	0.688
Hemoglobin	1.000 [1.000–1.000]	0.669
Thrombocytes	0.990 [0.981–0.998]	0.019
Leucocytes	1.110 [0.989–1.246]	0.076
Lymphocytes	0.955 [0.810–1.126]	0.585
Neutrophils	1.157 [1.010–1.324]	0.035
Serum potassium	1.372 [0.622–3.026]	0.433
Serum sodium	1.067 [0.956–1.191]	0.248
Errythrocyte sedimentation rate	0.969 [0.934–1.006]	0.096
Fibrinogen	0.996 [0.992–1.001]	0.087
C-reactive protein	1.000 [0.995–1.006]	0.945
Creatinin	1.310 [0.686–2.504]	0.413
Urea	1.012 [0.989–1.035]	0.312
NT-proBNP	1.000 [1.000–1.000]	0.060
Creatine kinase	1.000 [1.000–1.000]	0.892
Creatinine increase compared to baseline	5.978 [1.336–26.752]	0.019

Abbreviations: CI, confidence interval; OR, odds ratio. Other abbreviations: as in Table 1.

**Table 3:** Multivariate logistic regression analysis for ICU stay ≥ 7 days.

Parameter	OR [95% CI]	*p* Value
Cardiopulmonary bypass time	0.985 [0.964–1.006]	0.153
Aortic cross-clamp time	1.046 [1.014–1.080]	0.005
Chronic obstructive pulmonary disease	0.237 [0.025–2.228]	0.208
Postoperative thrombocyte count	0.985 [0.970–1.001]	0.061
Postoperative neutrophil count	1.172 [0.990–1.386]	0.065
Creatinine increase compared to baseline	6.560 [0.879–48.963]	0.441

Abbreviations: as in Table 2.

**Table 4:** Univariate logistic regression analysis for in-hospital mortality.

Parameter	OR [95% CI]	*p* Value
Demographic and Clinical		
Age	1.017 [0.957–1.081]	0.581
Sex	1.429 [0.426–4.792]	0.564
Arterial hypertension	0.687 [0.068–6.970]	0.751
Emergency surgery	0.082 [0.022–0.298]	<0.001
Preoperative		
Left ventricular ejection fraction	0.991 [0.850–1.154]	0.903
Total bilirubin	0.088 [0.001–12.143]	0.334
Alanine transaminase	0.972 [0.872–1.038]	0.605
Aspartate transaminase	0.997 [0.961–1.035]	0.875
Hemoglobin	1.000 [0.999–1.001]	0.875
Thrombocytes	1.004 [0.984–1.024]	0.721
Leucocytes	0.956 [0.573–1.593]	0.863
Lymphocytes	0.566 [0.032–9.983]	0.698
Neutrophils	0.604 [0.129–2.826]	0.522
Serum potassium	0.546 [0.048–6.187]	0.625
Serum sodium	0.936 [0.729–1.202]	0.603
C-reactive protein	0.998 [0.866–1.151]	0.981
Creatinin	0.933 [0.196–4.433]	0.930
Urea	1.032 [0.982–1.085]	0.211
Creatine kinase	0.996 [0.987–1.004]	0.338
Intraoperative		
Cardiopulmonary bypass time	1.012 [1.005–1.019]	0.001
Aortic cross-clamp time	1.010 [1.001–1.018]	0.021
Postoperative		
Vasopressors at ICU admission	0.154 [0.039–0.603]	0.007
Inotropes at ICU admission	0.603 [0.183–1.985]	0.405
Left ventricular ejection fraction	1.346 (0.811–2.235)	0.250
ICU length of stay	1.095 [1.035–1.158]	0.001
Albumin	0.042 [0.000–6.391]	0.217
Direct bilirubin	0.007 [0.000–1.338]	0.503
Total bilirubin	0.039 [0.000–80.817]	0.405
Alanine transaminase	0.875 [0.699–1.094]	0.241
Aspartate transaminase	0.966 [0.876–1.064]	0.480
Hemoglobin	1.000 [0.997–1.003]	0.951
Thrombocytes	0.920 [0.816–1.037]	0.172
Leucocytes	1.171 [0.823–1.664]	0.380
Lymphocytes	0.879 [0.180–4.295]	0.873
Neutrophils	1.208 [0.851–1.716]	0.290
Serum potassium	1.756 [0.133–23.218]	0.669
Serum sodium	1.095 [0.639–1.879]	0.740
Errythrocyte sedimentation rate	0.900 [0.698–1.161]	0.419
Fibrinogen	0.996 [0.982–1.011]	0.614
C-reactive protein	1.033 [0.987–1.082]	0.163
Creatinin	1.548 [0.429–5.591]	0.505
Urea	1.026 [0.976–1.078]	0.323
NT-proBNP	1.000 [1.000–1.000]	0.105
Creatine kinase	0.999 [0.993–1.005]	0.679

Abbreviations: as in the other tables.

**Table 5:** Multivariate logistic regression analysis for in-hospital mortality.

Parameter	OR [95% CI]	*p* Value
Cardiopulmonary bypass time	1.030 [1.003–1.057]	0.030
Aortic cross-clamp time	0.965 [0.934–0.997]	0.034
Emergency surgery	0.043 [0.002–0.863]	0.040
Vasopressors at ICU admission	0.001 [0.001–1.000]	0.996
ICU length of stay	0.149 [0.009–2.462]	0.183

Abbreviations: as in the other tables.

## Data Availability

The data presented in this study are available on request from the corresponding author, following the approval from the University of Medicine and Pharmacy of Craiova, Romania. The data are not publicly available due to privacy restrictions.

## Abbreviations

The following abbreviations are used in this manuscript:

AUC	Area under the curve
AXCT	Aortic cross-clamp time
CPBT	Cardiopulmonary bypass time
E-CABG	European Multicenter Study on Coronary Artery Bypass Grafting
EuroSCORE	European System for Cardiac Operative Risk Evaluation
ICU	Intensive care unit
IQR	Interquartile range
ROC	Receiver operator characteristics
STS	Society of Thoracic Surgeons
